# Voiding Dysfunction in Transgender Patients: What We Know and What We Do Not Know

**DOI:** 10.1007/s11934-024-01234-4

**Published:** 2024-11-05

**Authors:** Gabriela Gonzalez, Jennifer T. Anger

**Affiliations:** 1 Department of Urology, Davis School of Medicine, University of California, Sacramento, CA USA; 2 Department of Urology, San Diego School of Medicine, University of California, La Jolla, CA USA

**Keywords:** Genital reconstructive surgery, Gender affirming care, Vaginoplasty, Metoidioplasty, Phalloplasty, Urethral stricture, Urethrocutaneous fistula, Vaginal remnant, Voiding dysfunction

## Abstract

**Purpose of Review:**

Transgender and non-binary patients (TGNB) undergoing gender affirming genital surgery may experience perioperative voiding dysfunction. This review aims to outline and analyze literature about gender affirming pelvic surgery urinary complications, evaluation, and treatment.

**Recent Findings:**

If a patient is seeking bottom surgery, then urinary goals and pre-operative symptoms should be discussed with respect to variable post-operative outcomes. Urologists should also be aware of the effect that gender affirming hormone therapy has on urinary symptoms. Urethral strictures and urethrocutaneous fistulae occur after feminizing and masculinizing procedures and may manifest as LUTS. Although there is no standardized approach for managing post-operative voiding issues, we present available options.

**Summary:**

The evaluation of TGNB patients is ideally affirming and tailored to the patient. Long-term urinary and voiding outcomes measurements after vaginoplasty and phalloplasty are also needed, as current validated questionnaires do not capture these symptoms well in TGNB patients.

## Introduction

Transgender people have a gender identity that is incongruent with their assigned sex at birth, whereas non-binary individuals’ gender identity is neither completely male nor female. About 1.3 million (0.5%) adults identify as transgender in the United States [1]. Transgender and nonbinary (TGNB) patients experience significant barriers to seeking medical care, and regardless of their transition status, many individuals may alter their voiding habits due to fear of discrimination, violence, or lack of inclusivity in public restrooms. Respondents of the 2015 United States Transgender Survey documented being interrogated in restrooms (25%), denied restroom access (10%), and harassed or assaulted (12%) [2]. Transgender men (assigned female at birth, identify as men) are more likely than transgender women (assigned male at birth, identify as women) or non-binary individuals to avoid public restrooms [2].

Given the inherent gendered nature of many genitourinary conditions, creating an inclusive assessment and treatment plan for voiding dysfunction can be difficult [3]. This review covers important considerations when treating voiding dysfunction in TGNB individuals. When evaluating a TGNB patient, urologists should distinguish if they have undergone or are interested in future genital gender affirming surgery and determine where the patient is in their transition. Addressing LUTS after the anatomical changes associated with reconstructive surgery requires understanding of common post-operative sequelae for feminizing and masculinizing procedures. There are small, individual institution series analyzing voiding dysfunction after genital surgery. Kuhn et al. conducted a cross-sectional analysis of 52 transgender women who had affirming pelvic surgery and found stress urinary incontinence (SUI) and urinary urgency to be 23% and 24.6%, respectively [4]. An earlier study by Kuhn et al. surveyed 18 transgender women who underwent penile inversion vaginoplasty using the King’s Health Questionnaire. They demonstrated a 33% incontinence rate, primarily due to SUI and overactive bladder (OAB), which the authors suggest might be secondary to surgery, pudendal nerve damage, hormonal changes, and/or age-related progression [5]. Preoperative counseling, discussion of functional goals such as standing or sitting to void, cosmesis, and coital preferences must be addressed, as all these factors can be affected by voiding dysfunction.

### Considerations in Patients who have not Undergone Genital Affirming Genital Procedures

In general, urologists should take into account each patient’s anatomy and follow established urologic societal guidelines for the workup of common voiding conditions such as SUI, overactive bladder (OAB), pelvic pain, and benign prostatic hyperplasia (BPH). If patients are considering future gender affirming pelvic surgery, then treatment of LUTS should be discussed beforehand. For example, if a transgender man suffers from stress urinary incontinence (SUI) and is considering future masculinizing surgery, then pursuing a mid-urethral sling may be better to do first. This way, there is still access to the urethra in the event that a sling revision/takedown is needed.

Transgender men who have not undergone genital-affirming surgery and are taking testosterone hormone therapy may present with pelvic pain and irritative and voiding symptoms due to vulvovaginal atrophy [6]. Testosterone can cause significant atrophy, regardless of age. If a transgender man presents which such symptoms, an exam is warranted, but this can consist of a simple external inspection in order not to exacerbate gender dysphoria. Tordoff et al. conducted a cross-sectional analysis of 1,219 transgender and gender-diverse patients, which included a subset of patients on testosterone therapy, to investigate sexual function associated with vaginal atrophy. Almost half of the patients were treated with testosterone, and 64.6% reported genital pain, which was primarily located in the vagina or frontal genital introitus (52.2%) [7]. There are gaps in the literature regarding the role of vaginal estrogen in the hormonally treated transgender man. Extrapolating from the vaginal estrogen data for cisgender women, the use of topical estrogen does not increase serum estrogen levels and can help address LUTS in transgender men due to vulvovaginal atrophy. However, not all transgender men are willing to place a cream vaginally, as this process can exacerbate gender dysphoria.

Feminizing hormone therapy may also cause or exacerbate LUTS. The diuretic effects of spironolactone (used as an anti-androgen) can manifest as increased urinary frequency and urgency urinary incontinence. A study investigating whether estradiol is associated with LUTS in transgender women found that 81.4% of patients reported moderate incontinence using the International Prostate Symptom Score (IPSS) questionnaire [8]. Of note, this questionnaire has only been validated in cisgender men, but the findings shed light on a possible relationship between LUTS and gender affirming hormones. Patients who undergo orchiectomy may stop taking antiandrogen therapy. Although BPH could also cause LUTS in transgender women who have not undergone prior prostate surgery, it would be less of a factor in women who have been on gender-affirming hormone therapy (GAHT) from a young age. These women are less likely to have developed BPH than a woman starting hormones in mid to late adulthood.

### Voiding Dysfunction after Vaginoplasty

In a classic penile inversion vaginoplasty, the penis is disassembled and a neoclitoris created from the glans, the urethra is shortened and a vaginal canal is created between the urethra/prostate and rectum. The upper part of the vaginal canal is usually made of scrotal skin after months of electrolysis, and may be augmented with peritoneum as needed [9]. Vaginoplasty using the right or left colon is performed, based on surgeon and/or patient preference. Usually, colon vaginoplasty is reserved for women who may have had a prior complication/graft loss after penile inversion vaginoplasty.

Common urinary symptoms post vaginoplasty are anterior spraying of urine, irritative symptoms, incontinence, urinary tract infection (UTI), and urinary retention [10]. Urinary stream spraying can be due to an anteriorly positioned urethra, though some spraying is considered normal, as it is in cisgender women [11]. Urine deflection may improve after 3–6 months. However, if persistent, a surgical revision may be necessary by performing further periurethral dissection, allowing the urethral meatus to shift posteriorly to prevent a spraying urine stream. Patients with urine stream deviation may also endorse vaginal bulging during sexual arousal. This may require debulking of additional urethral spongiosum tissue [12]. Labial adhesions or bands may also cause a diminished or deflected urinary stream [13].

Bothersome obstructive voiding symptoms occur in about 14% of patients due to meatal stenosis (stricture) after vaginoplasty [14]. One retrospective single-center study of 339 transwomen patients post penile inversion vaginoplasty identified a 40% rate of meatal stenosis, 7% of whom developed acute urinary retention [15]. Urethral meatus narrowing can be surgically repaired with a wide meatotomy or a Y-V plastic reconstruction. About 15% of patients may require a secondary intervention due to a recurrence of stenosis [16]. Patients can also develop urethrovaginal fistulas from the proximal urethral pressure associated with a distal meatal stenosis.

Urinary retention in the immediate postoperative setting is usually self-limiting, and prior studies have reported a rate of 9–13% [17, 18]. Our voiding trial usually starts postoperative day five, when vaginal packing is removed and the patient begins to perform vaginal dilation. If the patient cannot void, the catheter is replaced for one week, and the patient can dilate with the foley in situ. However, some patients cannot tolerate dilating with the presence of a catheter, so the vaginal canal is re-packed, and the patient returns to the clinic for a voiding trial. Additional measures such as minimizing unnecessary narcotic medications, preventing constipation, and promoting ambulation can also help manage urinary retention.

Although the rates of urinary urgency and stress incontinence have been reported to range from 24.6% to 33% after vaginoplasty in two small-scale series, there are currently no standardized tools to quantify and follow these long-term outcomes.[4, 5]. SUI in transgender women after vaginoplasty may be exacerbated by a history of treatment of prostate cancer, LUTS, or trauma. Data is lacking regarding SUI management after vaginoplasty, which involves excising the distal urethra up to the bulb, therefore, making the later use of slings and artificial urinary sphincters (AUS) challenging. Though bulking agents are not routinely used in cisgender males with SUI, one surgeon has anecdotally reported success with transurethral bulking of the bladder neck for transgender women with SUI after vaginoplasty [19]. Alternatively, autologous or biological slings and AUS placed at the bladder neck can be considered.

Urinary tract infections (UTIs) may cause symptoms such as dysuria, frequency, urgency, or signs of sepsis. Postoperative UTIs have been reported at high rates (32%) after vaginoplasty, likely due to the shorter distance between the new urethral meatus and the bladder [20]. Patients should only be treated if they are symptomatic. As in cisgender women, antibiotic treatment should be culture-directed [21].

Although an adequate wide dissection of the pelvic floor muscles is necessary to create an appropriate neovaginal introitus width, there can be associated pelvic pain in up to 20% of patients [16, 17]. Spasticity of the pelvic floor can also contribute to pelvic pain postoperatively [19]. These symptoms can make vaginal canal dilation challenging, which is critical to preventing canal stenosis. Patients may also report dyspareunia and changes in genital sensation. These symptoms warrant an early referral to a pelvic floor physical therapist to assist with dilation. Patients who experience symptoms refractory to dilator therapy may warrant a dilation under anesthesia, which can be combined with injection of onabotulinum toxin into the pelvic floor muscles.

### Neovagina Prolapse

The prevalence of prolapse after vaginoplasty ranges from 1–2% [14, 16]. A small series by Kuhn et al. of 52 transwomen series who underwent vaginoplasty (49 penile inversion and 3 sigmoid vaginoplasty procedures) identified neovaginal prolapse stage II or greater in 7.5% of patients [4]. Surgical prolapse repair was performed in 3.8% of patients. The authors did not stratify the rate of symptomatic prolapse by index vaginoplasty procedure approach. Although there is limited literature, sacrospinous fixation during the initial vaginoplasty has been reported to prevent prolapse [14]. Postoperatively, when prolapse occurs, studies describe an abdominal sacrocolpopexy repair [22, 23]. Overall this complication is rare such that vaginal suspension procedures are not commonly performed at the time of vaginoplasty. However, it is a more common occurrence after colon vaginoplasty, though this usually consists of a minor degree of prolapse that can be addressed from a perineal approach.

### Masculinizing Procedures

Voiding while in a standing position is a significant goal for many transgender men who undergo a metoidioplasty with urethral lengthening or phalloplasty. Metoidioplasty involves creating a neophallus from a testosterone-virilized clitoris, usually after at least two years of testosterone therapy. Patients have the choice of undergoing a metoidioplasty with “urethral lengthening” which involves tubularization of labia minora flaps (with or without buccal graft, depending on the technique used) to connect the distal neourethra to the native proximal urethra. This allows for the man to void standing, though it is not always a guarantee. Usually, metoidioplasty is performed with a concomitant vaginectomy and scrotoplasty made from labia majora flaps. However, depending on patient coital preferences, the vaginal canal can be spared, though with a higher risk of urethrocutaneous fistula formation. Some patients may opt for a “simple metoidioplasty,” which creates a phallus from the clitoris, but does not involve urethral lengthening, scrotoplasty, or vaginectomy. Anticipated neophallus length and the ability to void standing can be challenging to predict pre-operatively. A standing-to-void rate of 87–100% has been reported when lengthening of the native urethra is performed, though this rate is lower in obese men and men with a low clitoral response to testosterone [24–26].

Neourethral strictures can occur after metoidioplasty. If they occur in the early post-operative period (while one still has his suprapubic tube, usually the first 3–4 weeks after surgery), the tube is left until three months after surgery, after which the blood supply will be robust and a urethroplasty (with likely buccal graft) can be performed. Fistula is another common complication of urethral lengthening. Fistulae do not prevent suprapubic tube removal, but formal repair is similarly delayed for three months after surgery until the urethral blood supply has been well established.

Vaginectomy, also known as colectomy, is performed during the creation of the neophallus (metoidioplasty or phalloplasty). The procedure involves fulguration or full-thickness resection of the vaginal epithelium together with a colpocleisis, or vaginal canal closure. It is hypothesized that mobilization of pelvic floor muscles during the vaginectomy can predispose patients to develop pelvic floor disorders such as de novo OAB and SUI [27]. Combaz et al. conducted a retrospective study of 120 patients who underwent masculinizing (18 patients) or feminizing (102 patients) procedures from four different centers. They analyzed post void residuals, bothersome urinary symptoms using the Visual Analogue Scale (VAS) and measured pelvic floor force with the revised Oxford Grading Scale [27]. The VAS identified 44 patients with urinary problems and all had multichannel urodynamics performed. Eight of the 44 patients underwent masculinizing procedures. All transgender men underwent a hysterectomy and oophorectomy, while six had a phalloplasty with urethral lengthening. The rates of LUTS among the eight transgender men included OAB (25%), SUI (38%), and post-void dribbling (37%). Patients who were diagnosed with OAB (15/44) without anatomical issues were treated with anticholinergics or mirabegron. Two patients experienced medication-refractory OAB and subsequently had 200 units of intravesical botulinum toxin administered. Half of the total patients experiencing SUI (12/44) improved with pelvic floor physical therapy, and the other six patients underwent transurethral administration of Bulkamid.

Superficial fulguration of the vaginal epithelium compared to excision can cause the formation of vaginal remnants [12, 24]. These remnants can communicate with the urethra and become incorporated as diverticula, leading to fistula formation in the presence of a distal obstruction that transmits pressure to the previously obliterated space. About 50% of patients with a neourethral stricture will have a persistent vaginal cavity [28]. A mucocele can form if the vaginal remnant does not communicate with the urinary tract. Patients may complain of obstructive voiding symptoms, high-volume post-void dribbling, perineal pain or fullness, and recurrent urinary tract infections [29]. Diagnostic evaluation may include flexible cystoscopy, MRI imaging, and assessment for urethral stricture [26]. Obliteration of the vaginal remnant can be done via a perineal approach or transabdominal with a robotic-assisted laparoscopic approach [29].

### Phalloplasty

Phalloplasty involves complex reconstructive procedures combining phallic shaft creation, urethroplasty, perineal urethral lengthening, perineoplasty, scrotoplasty, and staged glansplasty. There are various single-stage versus staged surgical techniques utilizing different shaft donor sites. The most common technique is a radial forearm free flap. An anterolateral thigh flap can also be used for men who do not wish to use their forearm or whose forearm may be too small for adequate phallus size. The donor sites are then covered with a split thickness skin graft, usually taken from the thigh.

The reconstructed phallic urethra is prone to strictures, particularly at the anastomosis between the pars fixa (the urethra constructed from labia minora) and the pendulous (phallic) urethra or throughout the phallic urethra [26]. A retrospective series of 22 patients undergoing post-phalloplasty stricture management identified 15% of strictures at the meatus, 28% in the pendulous urethra, 41% between the pars fixa and pars pendulans, 13% in the pars fixa, and 8% throughout several areas [30]. Management of neophallus stricture ranges from temporary catheter dilation and endoscopic interventions, including dilation or direct visualization internal urethrotomy (DVIU) to urethroplasty. In a retrospective review, Lumen et al. analyzed outcomes of endoscopic cold-knife incision for strictures shorter than 3 cm in post-phalloplasty patients [30]. They reported a success rate of almost 44% at a mean of 51 months for the first DVIU intervention. Our preferred management of early post-operative strictures is a gentle balloon dilation. Early post-operative stricture development was identified to be a significant predictor of endoscopic treatment failure [30]. Patients who want to avoid further reconstructive surgery or who have failed multiple repeat repairs can consider a perineal urethrostomy. As with metoidioplasty, distal obstruction from stricture disease can lead to proximal fistula formation. The incidence of urethrocutaneous fistula ranges from 15–70% [31]. Patients may report penoscrotal urine leakage along the anastomotic site between the pendulous urethra and the pars fixa or between the pars fixa and native urethra [31]. Although there is no standardized approach to fistula management, principles include complete excision of the fistula tract with a multi-layer flap closure to avoid overlapping suture lines. Certain fistula tracts may spontaneously close without intervention within 2–4 months, but fistulas that are large or present at multiple sites require surgical revision [12]. Patients who chose a canal-sparing approach have a 42% greater risk of fistula formation, likely due to the lack of vascularized vaginal tissue flaps [32]. However, like with metoidioplasty, these are usually amenable to a delayed repair.

Patients who undergo phalloplasty may also report post-void dribbling, which requires manual massaging of urine out of the meatus [33]. The dribbling results from the phallus not having bulbospongiosus muscular support to aid with micturition. In addition, if men do not have adequate electrolysis from the forearm (or thigh), hair can re-grow in the neourethra, leading to infections, stone formation, and voiding difficulty.

## Conclusions

Although our comprehension of diagnosing and treating voiding dysfunction after affirming pelvic surgery is growing, the field remains in in its infancy. The transition between natal anatomy and post-surgical anatomy can cause a wide range of urinary symptoms and impact quality of life. Accurately capturing short- and long-term outcomes with questionnaires validated in TGNB patients is greatly needed.

## Key References

- Wilson B, and Meyer IH. Nonbinary LGBTQ Adults in the United States. Available at https://www.williamsinstitutelawuclaedu/wp-content/uploads/Nonbinary-LGBTQ-Adults-Jun-2021pdf2021.
  - This research brief provides general statistics about transgender and non-binary patients in the United States.
- Gonzalez G, Anger J. Creating an Inclusive Urology Practice. Curr Bladder Dysfunct Rep. 2023;18(2):131–8.
  - This review article provides an overview of common terminology among gender diverse patients and suggests affirming medical practices for urologists.
- Tordoff DM, Lunn MR, Chen B, Flentje A, Dastur Z, Lubensky ME, et al. Testosterone use and sexual function among transgender men and gender diverse people assigned female at birth. Am J Obstet Gynecol. 2023;229(6):669.e1-.e17.
  - This article provides an overview of patient reported changes while on testosterone.
- Jun MS, Gonzalez E, Zhao LC, Bluebond-Langner R. Penile Inversion Vaginoplasty with Robotically Assisted Peritoneal Flaps. Plast Reconstr Surg. 2021;148(2):439–42.
  - A primer for penile inversion vaginoplasty steps.
- Fascelli M, Sajadi KP, Dugi DD, Dy GW. Urinary symptoms after genital gender-affirming penile construction, urethral lengthening and vaginectomy. Transl Androl Urol. 2023;12(5):932–43.
  - A comprehensive review of voiding symptoms after gender affirming pelvic surgery.

## Data Availability

No datasets were generated or analysed during the current study.
